# A survey of enabling technologies in synthetic biology

**DOI:** 10.1186/1754-1611-7-13

**Published:** 2013-05-10

**Authors:** Linda J Kahl, Drew Endy

**Affiliations:** 1 Bioengineering Department, Stanford University, Y2E2 Room 269C, 473 Via Ortega, Stanford, CA, 94305-4201, USA

**Keywords:** Synthetic biology, Biological engineering, Enabling technologies, Survey, Intellectual property rights, Licensing, Regulation

## Abstract

**Background:**

Realizing constructive applications of synthetic biology requires continued development of enabling technologies as well as policies and practices to ensure these technologies remain accessible for research. Broadly defined, enabling technologies for synthetic biology include any reagent or method that, alone or in combination with associated technologies, provides the means to generate any new research tool or application. Because applications of synthetic biology likely will embody multiple patented inventions, it will be important to create structures for managing intellectual property rights that best promote continued innovation. Monitoring the enabling technologies of synthetic biology will facilitate the systematic investigation of property rights coupled to these technologies and help shape policies and practices that impact the use, regulation, patenting, and licensing of these technologies.

**Results:**

We conducted a survey among a self-identifying community of practitioners engaged in synthetic biology research to obtain their opinions and experiences with technologies that support the engineering of biological systems. Technologies widely used and considered enabling by survey participants included public and private registries of biological parts, standard methods for physical assembly of DNA constructs, genomic databases, software tools for search, alignment, analysis, and editing of DNA sequences, and commercial services for DNA synthesis and sequencing. Standards and methods supporting measurement, functional composition, and data exchange were less widely used though still considered enabling by a subset of survey participants.

**Conclusions:**

The set of enabling technologies compiled from this survey provide insight into the many and varied technologies that support innovation in synthetic biology. Many of these technologies are widely accessible for use, either by virtue of being in the public domain or through legal tools such as non-exclusive licensing. Access to some patent protected technologies is less clear and use of these technologies may be subject to restrictions imposed by material transfer agreements or other contract terms. We expect the technologies considered enabling for synthetic biology to change as the field advances. By monitoring the enabling technologies of synthetic biology and addressing the policies and practices that impact their development and use, our hope is that the field will be better able to realize its full potential.

## Background

Synthetic biology is an emerging, interdisciplinary field that aims to make the design, construction, and optimization of biological systems easier and more reliable. Advances in synthetic biology will deepen our understanding of how biological systems work, and should enable faster and cheaper development of useful medicines, chemicals and materials, new means for information processing and data storage, and sources of food and energy that could help promote human health and preserve the environment. Although the field of synthetic biology is relatively young, there have already been promising advances in engineering microorganisms to produce important drugs [1,2], exploring strategies for biofuel production [3,4], and designing and DNA-based information storage [5,6] and genetically-encoded communications and processing systems [7,8].

As is true for many emerging fields of research, realizing the full potential for constructive applications of synthetic biology will require not only the continued development of enabling technologies but also the implementation of policies and practices to ensure that these technologies remain accessible to those working in basic and applied research. The enabling technologies for synthetic biology can be defined, broadly, as any reagent or method that, alone or in combination with associated technologies, provides the means to generate any new research tool or application in synthetic biology. Because the field of synthetic biology spans a wide range of disciplines – from engineering and biology to mathematics and computer science – the technologies considered “enabling” by synthetic biology researchers may be expected to cover a broad range, depending on the focus and nature of the research. Monitoring the enabling technologies of synthetic biology is an important step towards understanding the needs, abilities and accomplishments of this diverse research community.

Here, we conducted a survey among a self-identifying community of practitioners engaged in synthetic biology research to obtain their opinions and experiences with technologies that support the engineering of biological systems. The aim of this first study was to define a set of enabling technologies for the field of synthetic biology, with a focus on technologies used in research laboratories in both academia and industry. Our goal was to gather information about the technologies considered enabling by practitioners in the field so that we and others might better evaluate the landscape of synthetic biology and explore policies and practices that best promote continued innovation. For example, it is likely that useful applications of synthetic biology will embody multiple patented inventions and monitoring the enabling technologies of synthetic biology will facilitate the systematic investigation of the intellectual property rights coupled to those technologies. Investors and funders of synthetic biology research also may find this information useful in guiding funding decisions and establishing policies for the patenting and licensing of enabling technologies. Information gained from monitoring the enabling technologies of synthetic biology also may be useful in identifying technology trends, and could help government agencies and non-governmental organizations in crafting policy frameworks to address the safety and security concerns raised by synthetic biology research.

## Results

### Demographic data

During the period the survey was active, from August 31, 2012 to January 30, 2013 a total of 160 responses were received. Six responses were excluded because they did not contain answers to any of the substantive questions on technology use. Seventeen responses were from participants who answered “no” to Survey Question 11 that asked survey participants to indicate whether they considered themselves to be a synthetic biologist or to be engaged in basic or applied synthetic biology research or development. Responses from these 17 survey participants were analyzed separately, and the remaining 137 responses were used for most analyses.

Responses originated from the United States (121 responses, 88%) and ten other countries (16 responses, 12%). The distribution of responses from outside the United States was Australia (1), Canada (1), Germany (2), Israel (1), Italy (1), Japan (3), Mexico (1), Sweden (1), and the United Kingdom (5). Responses were received from researchers working exclusively in a non-commercial organization (n = 91, 66%), exclusively in a commercial organization (n = 39, 28%) and in both commercial and non-commercial organizations (n = 7, 5%). Among survey respondents working exclusively in a non-commercial organization, most indicated that they worked in a college or university (n = 64), research institution (n = 9), government laboratory (n = 1), were affiliated with both a college/university and research institution (n = 12) or were independent (n = 5). Among survey respondents working exclusively in a commercial organization, most were from small companies of fewer than 50 employees (n = 21) and the rest were from companies of more than 1000 employees (n = 11), fewer than 1000 employees (n = 3), and fewer than 250 employees (n = 4). Of the 7 survey respondents that worked in both commercial and non-commercial organizations, all worked in a small company of fewer than 50 employees as well as a college/university or research institution.

### Experience with the iGEM competition

Survey Question 2 asked participants to provide information about their experience as student or non-student participants of the International Genetically Engineered Machines (iGEM) competition (http://igem.org), a synthetic biology competition aimed at undergraduate students, high school students, and entrepreneurs. Of the 136 survey participants that responded to this question, 112 (82%) indicated that they did not participate in the iGEM competition as a student, 21 (15%) indicated that they previously participated in the iGEM competition as a student, and 3 (2%) indicated that they were a student currently participating in the iGEM competition.

In addition, 58 survey participants provided free-text responses describing their experience as non-student participants of the iGEM competition. Of these, 49 stated that they had advised or mentored iGEM teams or served as judges for the iGEM competition, 6 supported or sponsored iGEM teams, and 3 stated that they had other experience with the iGEM competition, including participating in organizing the software division, assisting in evaluation of the iGEM program, and reading iGEM research reports Thirteen of the 58 survey participants who provided free-text responses were also former students of the iGEM competition, while 45 had no prior experience with iGEM as students.

### Use of publicly available registries

Survey Question 3 asked participants to indicate whether they used publicly available registries to obtain natural or engineered biological materials or information. This question allowed survey participants to select from a list of publicly available registries and to write in any additional registries of which they were aware. The listed registries included publicly available collections of information (including DNA sequences) or tangible materials that could be used for synthetic biology research, including plasmids encoding specific biological functions, DNA-binding proteins, microorganisms and cell lines (hereinafter referred to as biological parts). Specifically, the publicly available registries initially listed included the Registry of Standard Biological Parts supporting the iGEM competition (iGEM Registry), the American Type Culture Collection (ATCC), Addgene, the Coli Genetic Stock Center (CGSC), the Synthetic Biology Engineering Resource Center (SynBERC) Registry, the Joint BioEnergy Institute Public Registry (JBEI-ICE Public), the European *Saccharomyces cerevisiae* Archive for Functional Analysis (EUROSCARF), the Agricultural Research Service NRRL collection (ARS/NRRL), the BIOFAB: International Open Facility Advancing Biotechnology (BIOFAB), the Dana-Farber/Harvard Cancer Center (DF/HCC) PlasmID Repository, the DNASU Plasmid Repository (DNASU), the Belgian Coordinated Collections of Micro-organisms (BCCM), and the Leibniz-Institut DSMZ - German Collection of Microorganisms and Cell Cultures (DSMZ).

**Figure 1:**
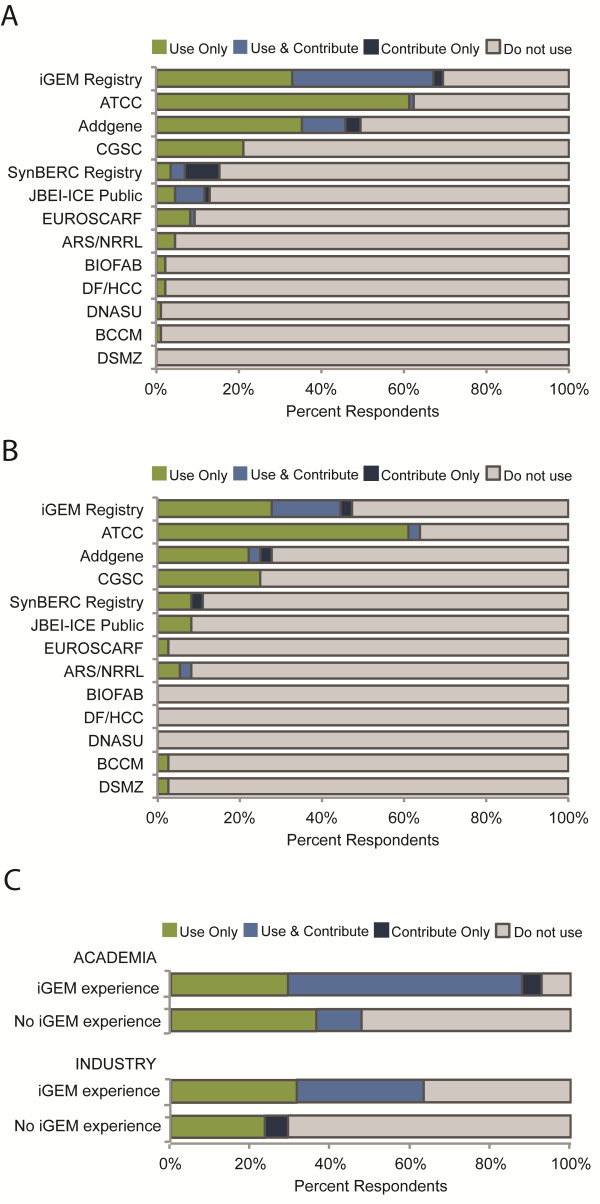
**Publicly available registries of natural or engineered biological materials or information.** (**A**) Percentage of synthetic biology researchers in academia that use biological parts from, or contribute parts to, publicly available registries. (**B**) Percentage of synthetic biology researchers in industry that use biological parts from, or contribute parts to, publicly available registries. (**C**) Impact of iGEM experience on use of the iGEM Registry by synthetic biology researchers in academia and industry.

The majority of synthetic biology researchers in academia (n = 88) used biological parts from or contributed parts to the iGEM Registry (n = 60, 68%) and the ATCC (n = 53, 60%), and many used or contributed to Addgene (n = 42, 48%) (Figure 1A). Other publicly available registries that were widely used among academic researchers included the CGSC (n = 18, 20%), the SynBERC Registry (n = 13, 15%), JBEI-ICE Public (n = 11, 12%) and EUROSCARF (n = 8, 9%). Fewer researchers in academia reported use or contribution of parts to the ARS/NRRL (n = 5), the BIOFAB (n = 2), the DF/HCC (n = 2), the DNASU (n = 2), the DSMZ (n = 1), and the BCCM (n = 1). Additional registries identified by academic researchers included the CyanoBase-Kazusa Genome Resources (http://www.ncbi.nlm.nih.gov/pmc/articles/PMC2808859) (n = 1), and the various yeast collections distributed by Invitrogen (n = 1).

Synthetic biology researchers in industry (n = 39) reported different usage rates for publicly available registries (Figure 1B). The majority of industry researchers used or contributed parts to the ATCC (n = 26, 67%), and many used or contributed parts to the iGEM Registry (n = 18, 46%). Other publicly available registries used by industry researchers included Addgene (n = 12, 31%), the CGSC (n = 9, 23%), the SynBERC Registry (n = 4, 10%), the JBEI-ICE Public (n = 3, 8%) and the ARS/NRRL (n = 4, 10%). Fewer industry researchers reported use or contribution of parts to the EUROSCARF (n = 2), the BCCM (n = 1), and the DSMZ (n = 2). Additional registries identified by industry researchers included the Keio/ASKA collection of *E. coli* strains (http://www.shigen.nig.ac.jp/ecoli/strain/top/top.jsp) (n = 1) and the pZ series expression vectors developed by Lutz and Bujard [9] (http://www.expressys.com) (n = 1).

Among the publicly available registries included in this survey, only the iGEM Registry showed a statistically significant difference in usage rates between researchers in academia and industry (p = 0.03). This difference was explored further by examining the impact of prior iGEM experience on use of the iGEM Registry (Figure 1C). Among synthetic biology researchers in academia, those having experience with the iGEM competition either as a student or non-student participant (e.g., advisors, judges, sponsors, etc.) were significantly more likely to contribute parts to or use parts from the iGEM Registry as compared to academic researchers without iGEM experience (93% and 46%, respectively, p < 0.00001). Similarly, industry researchers having experience with the iGEM competition were significantly more likely to use or contribute parts to the iGEM Registry than industry researchers lacking iGEM experience (65% and 26%, respectively, p = 0.02). Academic researchers having iGEM experience also were significantly more likely than industry researchers with iGEM experience to use or contribute to the iGEM Registry (93% and 65%, respectively, p < 0.01). No significant difference was observed in use of the iGEM Registry between academic and industry researchers without iGEM experience (46% and 26%, p = 0.17).

### Use of private registries

**Figure 2:**
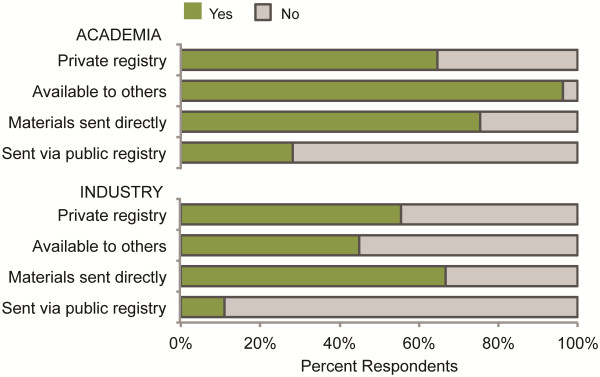
**Private registries of natural or engineered biological materials or information.** Percentage of synthetic biology researchers in academia and industry that maintain a private registry of biological parts, make these materials available to others, send materials to others directly, and send materials via a publicly available registry.

Survey Question 4 asked participants to indicate whether the laboratories or organizations in which they worked maintained a private registry of biological parts, and whether and how these parts were made available to others. Most synthetic biology researchers in academia reported that the laboratory or organization in which they worked maintained its own registry of biological parts (55/88, 62%) (Figure 2). Of these, the vast majority of academic researchers made these materials available to others outside their own laboratory (53/55, 96%). A significantly greater proportion of academic researchers sent parts directly to others (40/53, 75%) as compared to those that distributed parts through a publicly available registry (15/53, 28%) (p < 0.00001).

Similarly, most synthetic biology researchers in industry reported that the laboratory or organization in which they worked maintained its own registry of biological parts (21/39, 54%). However, fewer than half of industry researchers made these parts available to others (9/21, 43%). A significantly greater proportion of industry researchers also sent materials directly to others (6/9, 67%) as opposed to distributing parts through a publicly available registry (1/9, 11%) (p = 0.05).

A comparison of the use and distribution rates between academic and industry researchers revealed no statistically significant difference in the likelihood of maintaining a private registry of biological parts (p = 0.43). However, researchers in academia were significantly more likely to make parts available to others than researchers in industry (p < 0.000001).

### Favorite or most useful biological parts

A total of 43 participants responded to Survey Question 5, which was an open question asking respondents to list their favorite or most useful biological parts (Table 1). Biological parts that survey participants identified in more general terms included the Anderson promoter library (n = 4), in-house promoters (n = 2), three-color scaffold for monitoring gene expression (n = 1), dose-dependent promoters (n = 1), BIOFAB promoters (n = 1), fluorescent proteins (n = 9), colored proteins made at DNA2.0 (n = 1), all the working reporters in the iGEM Registry (n = 1), BioBrick vectors (n = 2), pRS vector series (n = 1), pZ vectors (n = 1), vectors (n = 1), high copy plasmid backbones from the iGEM Registry (n = 1), bicistronic design parts (n = 1), quorum sensing parts (n = 1), terminator variants (n = 1), and TetR (n = 2).

**Table 1:** Favorite or most useful biological parts from publicly available registries

**ID**	**Category**	**N**	**Description**
Addgene			
25712:pAKTaq	Plasmid	1	Bacterial expression vector encoding the DNA polymerase from *Thermus aquaticus*
Coli Genetic Stock Center			
CGSC #12119	Chassis	1	*E. coli* strain BW27783 bearing 9 known mutations
iGEM Registry			
BBa_J23100 series	Regulatory	8	BBa_J23100 through BBa_J23119 is a family of constitutive promoter parts that can be used to tune the expression level of constitutively expressed parts
BBa_B0034	RBS	4	RBS based on Elowitz & Liebler repressilator
BBa_B0015	Terminator	1	Double terminator including BBa_B0010 and BBa_B0012
BBa_C0062	Coding	1	luxR repressor/activator
BBa_E2050	Coding	1	derivative of mRFP1, yeast-optimized
BBa_F2620	Signaling	1	A signaling device whereby the input is 3OC_6_HSL and the output is PoPS from a LuxR-regulated operator
BBa_I15010	Coding	1	Chimeric Cph1 light receptor/EnvZ protein
BBa_I744210	Generator	1	TetR regulated LuxN-Tsr Chimeric Receptor B
BBa_J04450	Reporter	1	RFP coding device
BBa_J15001	RBS	1	strong synthetic *E. coli* RBS with SacI site
BBa_J153000	Plasmid Backbone	1	broad-host-range shuttle vector pPMQAK1 that provides ampicillin and kanamycin/neomycin resistance
BBa_J176005	Protein Domain	1	Codon optimized mCherry red fluorescent protein
BBa_J176006	Coding	1	Mammalian venus fluorescent protein
BBa_J176022	Protein Domain	1	Human codon-optimized AmCyan1 from pAmCyan1-C1
BBa_J33207	Reporter	1	lac promoter and lacZ
BBa_J61009	Plasmid	1	pAC-LuxGFP that places GFP under the wildtype Vibrio lux device
BBa_J64032	Device	1	pCASP SPI-1 Secretion Circuit
BBa_J85226	Composite	1	Kanamycin resistance (KanR)_off version of J85224
BBa_J176027	Regulatory	1	Constitutive cytomegalovirus promoter
BBa_J176122	Plasmid Backbone	1	pcDNA3.1 plus puromycin resistance
BBa_K566002	Regulatory	1	Biphasic switch
BBa_P1010	Generator	1	ccdB cell death gene
BBa_R0040	Regulatory	1	TetR repressible promoter

### Use of physical assembly standards and methods

**Figure 3:**
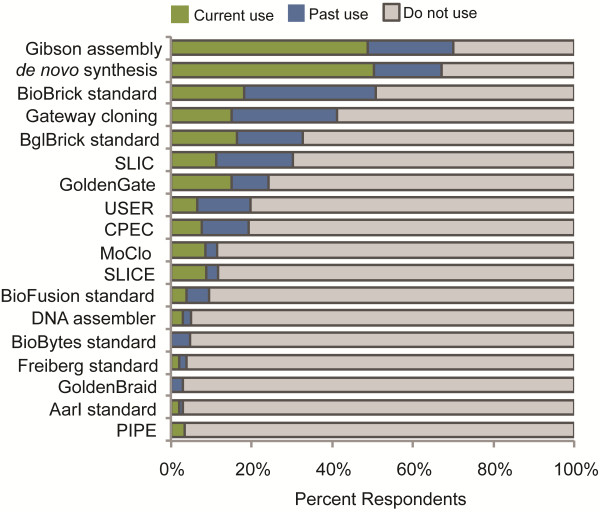
**Physical assembly standards and methods.** Current and past use of physical assembly standards and methods by synthetic biology researchers.

Survey Question 6 asked participants to indicate their current and past use of physical assembly standards and methods. This question allowed survey participants to select from a list of physical assembly methods and to write in any additional assembly methods that they used. Of the 134 survey participants that answered this question, most indicated that they currently use or previously have used the Gibson assembly method (48% current, 22% past) and *de novo* DNA synthesis (50% current, 18% past) (Figure 3). The original BioBrick standard (18% current, 31% past) and Gateway cloning (15% current, 26% past) were selected by a significant number of survey participants, although most indicated past use of these specific methods. Survey participants indicated lower overall usage rates for other physical assembly methods, including the BglBrick standard (16% current, 16% past), Sequence and Ligase Independent Cloning (SLIC) (14% current, 21% past), GoldenGate (16% current, 10% past), and others.

In addition to the physical assembly methods listed in Question 6, survey participants providing free-text responses to this question (n = 33) identified additional assembly methods that they used in the course of synthetic biology research (Table 2).

**Table 2:** Additional physical assembly methods identified by survey participants

**Physical Assembly Method**	**N**
Commercial/Proprietary	
Proprietary method, not specified	3
GeneArt Seamless Assembly	2
GeneArt High Order Assembly	1
Clontech In-Fusion HD cloning kit	1
Ginkgo assembly method	1
Invitrogen TOPO cloning	1
Non-Commercial	
Conventional PCR [10,11]	10
Yeast *in vivo* recombinational cloning [12]	5
Home-brew method, not specified	4
Restriction-site Associated DNA (RAD) assembly [13]	2
Anderson 2 antibiotic (2ab) assembly [14]	1
Inverse PCR [15]	1
Splicing by Overlap Extension (SOE) [16]	1
In development	
A new enzymatic, scarless synthesis and assembly technology	1
Extensions of BioBytes assembly standard [17]	1

### Use of measurement, functional composition and data exchange standards and tools

**Figure 4:**
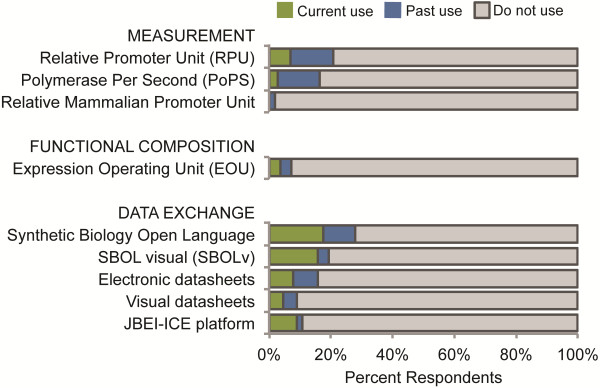
**Standards and methods for measurement, functional composition, and data exchange.** Current and past use of standards and methods for measurement, functional composition, and data exchange by synthetic biology researchers.

A total of 120 and 127 survey participants responded to Survey Questions 7 and 8, respectively, which asked respondents to indicate their current or past use of measurement tools, functional composition standards, and data exchange tools. These questions allowed survey participants to select from a list of tools and to write in any additional measurement, functional composition and data exchange tools that they used. Usage rates for measurement standards, functional composition standards, and data exchange standards were relatively low (Figure 4). Specifically, the number of survey respondents reporting current and past use were: Relative Promoter Unit (RPU) (7% current, 14% past), Polymerase Per Second (PoPS) (2% current, 14% past), Relative Mammalian Promoter Unit (RMPU) (0 current, 2% past), Expression Operating Unit (EOU) (4% current, 3% past), Synthetic Biology Open Language (SBOL) (18% current, 10% past), SBOL visual (SBOLv) (16% current, 3% past), JBEI-ICE repository platform (8% current, 2% past), electronic datasheets (7% current, 7% past), and visual datasheets (4% current, 4% past).

Additional measurement tools identified by survey participants included fluorescence reporter protein measurement (n = 4), Miller assay (n = 2), beta-galactosidase assay (n = 2), cell auto-fluorescence (n = 1), dual-luciferase reporter measurement (n = 1), comprehensive metabolite measurement (n = 1), comprehensive proteome (n = 1), specific mRNA or protein measurements (n = 1), RNAseq (n = 1), and qPCR (n = 1). Additional data exchange tools included custom laboratory management information systems (n = 2), JERM (n = 1), RightField (n = 1), Systems Biology Markup Language (SMBL) (n = 1), GoogleDocs (n = 1), and the GenBank file format (n = 1).

### Use of additional tools, reagents and methods

**Figure 5:**
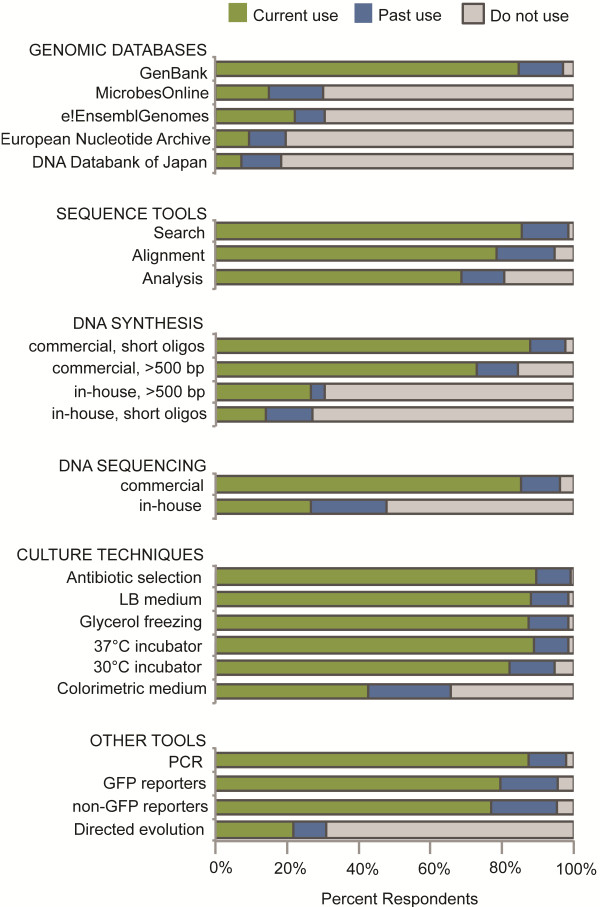
**Additional tools, reagents and methods.** Current and past use of genomic databases, sequence tools, DNA synthesis tools, DNA sequencing tools, culture techniques, and other tools, reagents, and methods by synthetic biology researchers.

Survey Question 9 asked participants to indicate their current and past use of additional tools, reagents, and methods. This question allowed survey participants to select from a list and to write in any other tool reagent or method that they considered enabling for the field of synthetic biology. Of the survey participants that responded to this question, the vast majority indicated that they used GenBank as their preferred genomic database (84% current, 12% past) and employed tools for search (84% current, 15% past), alignment (78% current, 16% past), and analysis (68% current, 13% past) of DNA sequences (Figure 5). Most survey participants used commercial DNA synthesis services for short oligos (86% current, 11% past) and gene-size fragments (71% current, 12% past), while fewer researchers used in-house DNA synthesis for short oligos (14% current, 15% past) and gene-size fragments (26% current, 44% past). The vast majority of survey participants indicated that they used established culture techniques, as well as other established technologies such as the polymerase chain reaction (PCR), green fluorescent protein (GFP), and non-GFP reporter molecules, while fewer researchers used newer techniques such as directed evolution (e.g., MAGE) (22% current, 10% past).

In addition to the tools, reagents and methods that were listed, survey participants providing free text responses (n = 23) identified additional technologies that they considered enabling for synthetic biology (Table 3).

**Table 3:** Additional technologies considered enabling for synthetic biology by survey participants

**Tool, Reagent or Method**	**Description (URL or reference)**	**N**
BioCyc	a collection of 1962 pathway and genome databases (http://biocyc.org)	1
Bioinformatics	the application of computational techniques to analyze the information associated with biomolecules on a large-scale (http://www.ncbi.nlm.nih.gov/About/primer/bioinformatics.html)	1
CAGE: Conjugative Assembly Genome Engineering	a technology that permits the hierarchical consolidation of modified genomic regions [18]	1
High Throughput Computing	the ability to run many copies of software at the same time across many different computers, reviewed in [19]	1
*in vitro* screens	tests for biological activity such as metal binding screens, electron uptake, and other enzymatic activity	2
IonTorrent	an approach to DNA sequencing that enables a direct connection between chemical and digital information and aims to place DNA sequencing within the reach of any laboratory or clinic [20]	1
EcoCyc	a database for *Escherichia coli* K-12 MG1655 (http://ecocyc.org)	1
Flow Cytometry	a technology that uses the principles of light scattering, light excitation, and emission of fluorochrome molecules to generate specific multi-parameter data from particles and cells in the size range of 0.5 μm to 40 μm diameter (http://crl.berkeley.edu/flow_cytometry_basic.html)	3
KEGG: Kyoto Encyclopedia of Genes and Genomes	a database resource for understanding high-level functions and utilities of the biological system, such as the cell, the organism and the ecosystem, from genomic and molecular-level information (http://www.genome.jp/kegg)	1
Mass spectrometry	a technology for targeted protein quantification, reviewed in [21]	2
MetaCyc	a database of nonredundant, experimentally elucidated metabolic pathways (http://metacyc.org)	1
Molecular biology technologies, generally	includes methods and reagents for creating competent cells, nucleic acid transfer, digestion, primer extension, ligation, assembly of DNA molecules, etc.	9
OptForce	an algorithm that identifies all possible metabolic interventions that lead to the overproduction of a biochemical of interest [22]	1
PDB: Protein DataBank	an information portal to biological macromolecular structures (http://www.rcsb.org/pdb/home/home.do)	1
Protein purification technologies	methods for purifying a protein of interest efficiently, reviewed in [23]	1
Recombineering	an *in vivo* method of genetic engineering applicable to chromosomal and episomal replicons in *E. coli*[24]	1
Robotic automation	use of robots for repetitive laboratory tasks such as pick and place, liquid and solid additions, heating, cooling, mixing, shaking, etc.	2
Single cell microscopy	a technology that enables visualization of gene expression with exquisite spatial and temporal sensitivity, reviewed in [25]	1
Standards, needed	includes standards for calibrating and sharing data from plate readers, standards for test, measurement and characterization, standards for documentation and sharing of biological modules, for example see Arkin, 2008 [26] and Endy, 2005 [27]	3
SOLiD	a next generation sequencing technology that allows identification of hundreds of millions of short RNAs in a sample in a single run [28]	1
Transcription Activator-Like (TAL) effector technology	a technology that allows proteins to be designed to specifically target and bind to a desired sequence of DNA [29]	1
UniProt: Universal Protein Resource	a collaboration between the European Bioinformatics Institute (EBI), the SIB Swiss Institute of Bioinformatics and the Protein Information Resource (PIR) that aims to provide a comprehensive resource for protein sequence and annotation data (http://www.uniprot.org)	1
Yeast *in vivo* recombination	methods for assembling large DNA constructs in the yeast *Saccharomyces cerevisiae,* for example see Gibson et al., 2008 [30] and Jaschke et al., 2012 [31]	1

### Use of software tools

**Figure 6:**
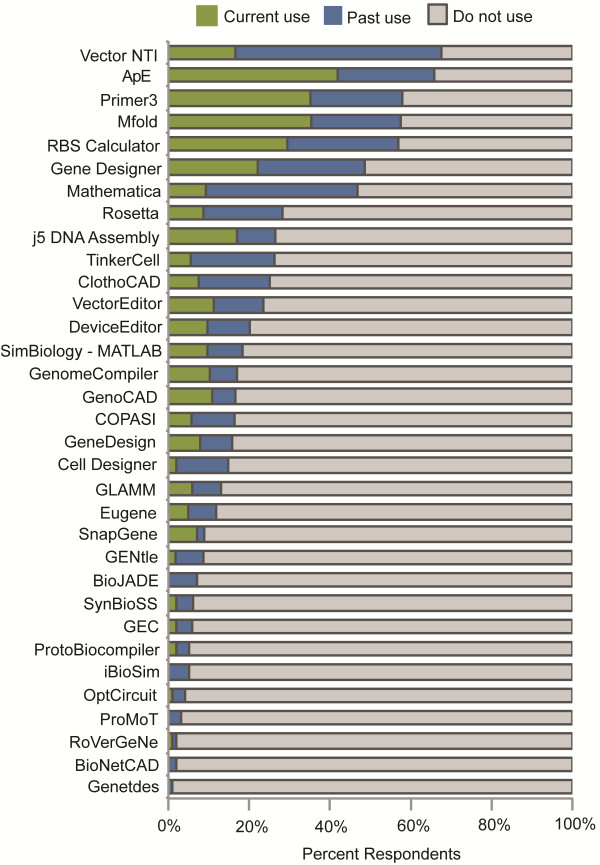
**Software tools.** Current and past use of software tools by synthetic biology researchers.

A total of 133 survey participants responded to Survey Question 10, which asked participants to indicate their current and past use of software tools. This question allowed survey participants to select software tools that were listed and to write in any additional software tools they used. The highest rates of current use were reported for ApE (41% current, 24% past), Primer 3 (33% current, 24% past), Mfold (34% current, 21% past), and the Ribosome Binding Site (RBS) Calculator (28% current, 28% past) (Figure 6). Vector NTI (17% current, 51% past), GeneDesigner (22% current, 27% past), and Mathematica (11% current, 35% past) were selected by a significant number of survey participants, although most indicated past use of these software tools. Lower but increasing-over-time usage rates were reported for the j5 DNA Assembly (16% current, 9% past), Genome Compiler (11% current, 6% past), and GenoCAD (10% current, 6% past) software tools. In addition to the software tools that were listed, survey participants providing free text responses (n = 36) identified additional software tools used in the course of their synthetic biology research (Table 4).

**Table 4:** Additional software tools used for synthetic biology research by survey participants

**Software tool**	**Description (URL)**	**N**
ABySS: Assembly By Short Sequences	a *de novo*, parallel, paired-end sequence assembler that is designed for short reads (http://www.bcgsc.ca/platform/bioinfo/software/abyss)	1
AlignDNA	a pairwise DNA alignment tool (http://www.geneinfinity.org/sms/sms_aligndna.html)	1
BLAST: Basic Local Alignment Search Tool	a program that finds regions of local similarity between biological sequences (http://blast.ncbi.nlm.nih.gov)	7
CentroidFold	a program that predicts an RNA secondary structure from an RNA sequence (http://www.ncrna.org/centroidfold)	1
CLC Genomics Workbench	a comprehensive and user-friendly analysis package for analyzing, comparing, and visualizing next generation sequencing data (http://www.clcbio.com/products/clc-genomics-workbench)	5
CodonCode Aligner	a program for sequence assembly, contig editing, and mutation detection (http://www.codoncode.com/aligner)	1
Cytoscape	an open source software platform for visualizing and integrating complex networks (http://www.cytoscape.org)	1
FastPCR	a program for PCR primer design (http://en.bio-soft.net/pcr/FastPCR.html)	1
Gene Construction Kit	a program for plasmid mapping (http://www.textco.com/gene-construction-kit.php)	1
Geneious	a program for handling and managing bioinformatics data (http://www.geneious.com)	7
Gibthon Ligation Calculator	a software tool for calculating reactant concentrations for DNA ligation (http://django.gibthon.org/tools/ligcalc)	1
JWS online	a tool for simulation of kinetic models from a curated model database (http://jjj.biochem.sun.ac.za)	1
Lasergene-DNAStar	comprehensive software for DNA and protein sequence analysis, contig assembly and sequence project management (http://www.dnastar.com)	7
Mascot	a search engine which uses mass spectrometry data to identify proteins from primary sequence databases (http://www.matrixscience.com/search_intro.html)	1
Mauve	a system for efficiently constructing multiple genome alignments in the presence of large-scale evolutionary events such as rearrangement and inversion (http://gel.ahabs.wisc.edu/mauve)	1
Merlin	a M.A.G.E. optimization tool developed by the Cross-disciplinary Integration of Design Automation Research group at Boston University (http://cidar1.bu.edu:8080)	1
OligoAnalyzer	software for comprehensive oligonucleotide analysis (http://www.idtdna.com/analyzer/Applications/OligoAnalyzer)	1
ORF Finder: Open Reading Frame Finder	a graphical analysis tool which finds all open reading frames of a selectable minimum size in a user’s sequence or in a sequence already in the database (http://www.ncbi.nlm.nih.gov/projects/gorf)	1
PaR-PaR	software that allows researchers to use liquid-handling robots effectively (http://prpr.jbei.org)	1
Pigeon	Synthetic Biology Open Language picture generator (http://cidar1.bu.edu:5801/pigeon1.php)	1
PlasMapper	software that automatically generates and annotates plasmid maps using only the plasmid DNA sequence as input (http://wishart.biology.ualberta.ca/PlasMapper)	1
PyMOL	a user-sponsored molecular visualization system for rendering and animating 3D molecular structures on an open-source foundation (http://pymol.org)	1
RNAstructure	a complete package for RNA and DNA secondary structure prediction and analysis (http://rna.urmc.rochester.edu/RNAstructure.html)	1
Serial Cloner	freeware with an intuitive interface that assists in DNA cloning, sequence analysis and visualization (http://serialbasics.free.fr/Serial_Cloner.html)	3
Sequencher	DNA sequencing software (http://genecodes.com/sequencher-features)	1
SSC: Stochastic Simulation Compiler	a tool for creating exact stochastic simulations of biochemical reaction networks (http://web.mit.edu/irc/ssc)	1
SWISS-PDB viewer (aka DeepView)	an application that provides a user friendly interface allowing analysis of several proteins at the same time (http://spdbv.vital-it.ch)	1
Synbiota	a platform of collaborative services to design, store, post, organize, access, or share information (https://mozillalabs.com/en-US/synbiota)	2
Velvet	a set of algorithms to manipulate de Bruijn graphs for genomic sequence assembly (http://www.ebi.ac.uk/~zerbino/velvet)	1

### Technology choices and self-identification as a synthetic biologist

Survey Question 11 asked participants whether they considered themselves to be a synthetic biologist or to be engaged in basic or applied synthetic biology research or development. Because this question was introduced on Day 6 of the survey, not all participants were able to respond to this question. A total of 58 survey participants had submitted responses prior to Day 6 and because all of these participants were students or postdoctoral fellows in the Endy or Smolke labs or researchers affiliated with SynBERC they were considered to be synthetic biology researchers for the purposes of the survey. Of the 96 survey participants that submitted responses on Day 6 and later, 79 answered “yes” and 17 answered “no” to this question. Responses from the 79 participants that affirmatively self-identified as synthetic biologists and the 58 participants that responded prior to Day 6 were grouped together for most analyses. Responses from the 17 participants that did not self-identify as synthetic biologists were analyzed separately.

Of the 17 participants that did not self-identify as synthetic biologists, 12 were from the US, 2 were from the UK, 1 was from Austria, 1 was from Norway, and 1 was from Portugal. Eleven participants worked exclusively in non-commercial organizations – 5 in a college or university, 2 in a research institution, 1 in both a college/university and research institution, and 3 were independent. The remaining 6 worked exclusively in commercial organizations – 3 in a company with more than 1,000 employees, 2 in a company of fewer than 50 employees, and 1 in a company of fewer than 250 employees. Twelve of the 17 participants had no experience with the iGEM competition, 1 had been a sponsor for iGEM teams, and 1 collaborated with an iGEM team as a DIY biologist.

Among the 17 participants who did not self-identify as synthetic biologists, two indicated that they used parts from the iGEM Registry (one was a former iGEM student, the other had no iGEM experience). In addition, some participants used parts from other publicly available registries, including the ATCC (n = 5), Addgene (n = 3), the ARS/NRRL (n = 2), the BIOFAB (n = 1), and the CGSC (n = 1). With regards to private registries, 4 participants (3 from academia, 2 from industry) indicated that the laboratories or organizations in which they worked maintained a private registry of biological parts. Of these, 3 made parts available to others (all from academia) – 3 by sending parts directly, and 1 by also distributing parts through a publicly available registry. None of these 17 participants reported current use of standards and methods for measurement, functional composition, or data exchange, although past use of the RPU (n = 1), electronic datasheets (n = 1), and visual datasheets (n = 1) was noted. Both current and past use was reported for other technologies covered in this survey, including physical assembly methods, software tools, and other tools, reagents, and methods (Table 5).

**Table 5:** **Technologies used by survey participants that did not self-identify as synthetic biologists (n** = **17)**

**Tool, reagent or method**	**Current use**	**Past use**	**Tool, reagent or method**	**Current use**	**Past use**
Physical assembly methods			Genomic database		
Gateway recombinatorial cloning	4	1	GenBank	10	3
*de novo* DNA synthesis	3	2	E!EnsemblGenomes	7	3
Gibson assembly	3	1	MicrobesOnline	3	1
Conventional restriction site-based cloning	2	0	European Nucleotide Archive	2	2
CPEC	1	2	DNA Databank of Japan	2	0
SLIC	1	1	Sequence tools		
PIPE	1	1	Search (e.g., BLAST)	12	3
USER	1	1	Alignment (e.g., ClustalW2)	10	1
InFusion cloning	1	0	Analysis (e.g., OligoCalc)	7	2
RAD assembly	1	0	Software tools		
Transfer PCR	1	0	ApE	6	1
Yeast *in vivo* cloning	1	0	Rosetta	4	1
BioBrick assembly standard	0	1	Vector NTI	3	5
Measurement, functional composition, data exchange			Primer3	3	3
RPU	0	1	Mathematica	2	5
Electronic datasheets	0	1	Mfold	1	2
Visual datasheets	0	1	Gene Designer	1	2
DNA synthesis			Blast	1	1
Commercial, short oligos	9	4	J5 DNA Assembly	1	1
Commercial, gene size (>500bp)	4	3	Vector Editor	1	1
In-house, gene-size(>500 bp)	3	2	GenoCAD	1	1
In-house, short oligos	1	4	Cell Designer	1	0
DNA sequencing			ClothoCAD	1	0
Commercial	8	3	iBioSim	1	0
In-house	2	4	Gene Design	1	0
Culture technique			ProtoBiocompiler	1	0
LB broth or agar	10	3	SimBiology	1	0
37°C incubator	10	3	SnapGene	1	0
Antibiotic selection	9	4	TinkerCell	1	0
Glycerol freezing	8	3	GenomeCompiler	0	1
30°C incubator	7	3	GEntle	0	1
Colorimetric medium	4	2	GLAMM	0	1
Other tools, reagents and methods			COPASI	0	1
PCR	9	5	DeviceEditor	0	1
GFP reporters	7	4	Lasegene-DNA Star	0	1
Non-GFP reporters	6	4	RBS Calculator	0	1
Directed evolution	3	2			

## Discussion

The emerging field of synthetic biology has captured the interest and energy of researchers from a variety of disciplines – biology, chemistry, computer science, engineering, and more. Our survey tapped the experiences and perspectives of this diverse research community in order to glean initial insights into the technologies that are considered enabling for the field by its practitioners. The results of the survey offer a first snapshot view of the technologies previously and now in use by synthetic biology researchers, and give a sense of the many and varied technologies that support work in synthetic biology.

One of our objectives in conducting this survey was to establish a set of technologies considered enabling for the field of synthetic biology, so that we and others might systematically investigate the intellectual property rights coupled to these technologies. For example, one overall consideration regarding enabling technologies and property rights is whether or not these technologies are accessible for use – either by virtue of being in the public domain or through legal tools such as non-exclusive licensing – to researchers in academic, government, and commercial organizations. The extent to which innovation in synthetic biology, and biotechnology more generally, may be impeded by broad foundational patents that cannot be licensed or patent thickets remains unclear [32-36]. Identifying the technologies to which wide, unrestricted access is needed to promote continued innovation in synthetic biology is an important step towards understanding the impact of patenting and licensing practices on access to the enabling technologies underlying this field.

Consistent with the postulate that past scientific achievements lay the foundation for future innovation, the results of the survey showed that many of the technologies that enable research in synthetic biology are well established and in the public domain. For many of these earlier technologies patent protection was either not sought or, even if patent protected, sufficient time has lapsed for the technologies to enter the public domain. For example, the vast majority of survey respondents reported use of bacterial cell culture technologies such as LB medium or glycerol freezing (Figure 5), yet these technologies were published in the scientific literature as early as the 1950’s [37-39] and are squarely in the public domain. Similarly, the vast majority of survey respondents reported use of PCR technology, yet elements of PCR technology have entered the public domain or will do so shortly. Specifically, foundational patents covering amplification methods (e.g., US 4,683,195 and EP 0 200 362 B), thermal cycling instruments (e.g., US 5,038,852 and EP 0 395 736 B), and thermostable DNA polymerases (e.g., US 4,889,818 and EP 0 258 017 B) have now expired. Although patents continue to be filed on improvements to PCR technologies, many subsequent patents such as those covering thermostable polymerases with enhanced activities (e.g. US 5,436,149 and US 5,556,772) have either expired or are due to expire within the next year.

Other more recently developed technologies used by synthetic biology researchers are currently patent protected or have patent applications pending, yet are accessible through non-exclusive licensing. For example, the majority of survey respondents reported current or past use of the Gibson assembly method [40] for *in vitro* physical assembly of DNA constructs (Figure 3). Several granted patents and pending patent applications are relevant to this method, including US Patents 7,723,077 and 7,776,532, US Applications 2010/0184187 and 2010/0311126, and International Application PCT/US2006/031214. Although the exclusive period for these patents is expected to extend through at least 2026, access to the Gibson assembly method has been made available through a non-exclusive licensing agreement between Synthetic Genomics, Inc. and New England BioLabs, Inc. [41]. As such, components may be purchased from New England BioLabs, Inc., albeit with significant restrictions that limit use to “internal research purposes for the sole benefit of the purchaser only” [42]. Those desiring additional rights to the Gibson assembly method, for example to manufacture commercial products, must contact Synthetic Genomics, Inc. directly.

Still other technologies that survey respondents consider enabling for the field of synthetic biology are heavily patent protected and the ability of researchers to access these technologies through licensing is less clear. For example, the majority of survey respondents indicated current or past use of GFP or non-GFP reporter molecules (Figure 5). Fluorescent proteins are commonly used as genetically encoded reporter molecules that enable researchers to observe the activity of particular genetic elements and biomolecules inside live cells or tissues. One of the foundational patents covering uses of GFP (US 5,491,084) is due to expire in September 2013. However, the exclusivity periods for other foundational patents on GFP and its uses are expected to continue for a number of years (e.g., US 5,741,668, US 6,146,826, EP 0 759 170 B1). Furthermore, there are literally hundreds, if not thousands, of issued patents covering variants of GFP and their uses. For example, a search of CAMBIA’s Patent Lens (http://www.patentlens.net) for the term “green fluorescent protein” in the same claim [i.e., Expert Search of (green near/2 fluorescent) and (fluorescent near/2 protein) and (green near/2 protein) in claims] yielded 770 granted US patents and 256 granted European patents. Similar searches for yellow fluorescent protein, red fluorescent protein, and blue fluorescent protein yielded over 400 patents granted in the US and Europe. The large number of patents covering variants of GFP and their uses presents a considerable challenge to synthetic biology researchers who wish to use fluorescent reporters in creating standards for characterizing biological parts and devices, such as the Relative Promoter Unit (discussed below). Some relief to navigating this thicket of patents may be available through negotiating a license agreement with Life Technologies or GE Healthcare. For example, the ATCC has recently announced that they have secured a license agreement that enables them to distribute GFP-containing biological materials to non-commercial and government researchers [43]. However, for-profit customers must have a separate license with Life Technologies or GE Healthcare to obtain and use these materials.

In addition to the observations noted above, the survey results indicate several trends for the use of technologies by synthetic biology researchers. The majority of survey respondents reported that they used biological parts from or contributed parts to publicly available registries (Figure 1) as well as private registries maintained within individual laboratories (Figure 2). Among the most widely used publicly available registries were the iGEM Registry, the ATCC, and Addgene. These registries, as well as many of the others listed in the survey, provide researchers with tangible materials (e.g., cultures, plasmids, and other reagents) as well as information relevant to the material (e.g., source, nucleic acid sequence, performance specifications). From a technical perspective, public registries are useful only to the degree that the biological materials contained within are reliable and accurately described. Towards that end, several publicly available registries have undertaken steps to curate the parts received and to verify nucleic acid sequence information. From an intellectual property perspective, access to materials from public registries is limited not only by considerations of patent protection, but also by the terms of material transfer agreements or other contracts that may govern the transfer of tangible materials. Registries of biological parts such as the SynBERC registry, the JBEI-ICE Public, and the BIOFAB currently provide information only. To the extent that the genetically-encoded materials described in these registries may be readily synthesized from the sequence information provided, use of these materials is limited primarily by considerations of patent protection. Although the SynBERC and JBEI-ICE Public registries currently indicate whether the material is “encumbered” or “not encumbered,” no other information is provided to assist researchers wishing to use these materials with identifying relevant patents. As for private registries, many survey respondents indicated that they shared materials with others and that they distributed materials by direct transfer as well as through publicly available registries. The types of materials maintained in private registries and the terms for their transfer were not queried in this survey, though considerations of patent protection and possibly additional contract terms would be relevant to use of materials from private registries as well.

Not all of the technologies queried in this survey were used by the majority of survey participants. Unlike the high usage rates reported for physical assembly standards and methods (Figure 3, Table 2), relatively few survey participants indicated current or past use of standards and methods for measurement, functional composition, and data exchange (Figure 4). One possibility that could account for such relatively low usage rates is that some types of standards and methods have been introduced only recently. For example, the EOU has been presented at meetings as early as 2010 [44], but an initial formal description and example applications of the EOU have only recently been published [45,46]. Another possibility that could account for the relatively low usage rate is that some types of standards and methods require tools that are not readily licensed by industry. For example, the RPU requires measurement of fluorescent reporter molecules [47] and it may be necessary to work through the patent thicket surrounding uses of GFP before widespread adoption of such standards is possible. Finally, it may also be that some technologies identified in this survey are useful to only a subset of survey respondents. The synthetic biology research community is comprised of diverse group of individuals with varied interests and goals. As such, the technologies that are considered vitally important for the work of some researchers may not be used at all by others.

To the extent that one or more patented technologies could eventually become widely adopted as a standard in the field, it may be advisable for the synthetic biology research community to consider creating more formal organizational structures and to articulate policies and best practices for the disclosure and licensing of patented technologies. Although standardization in synthetic biology is still at a relatively young stage [48,49], there are indications that the patent landscape is becoming quite complex [50]. To mitigate potential difficulties in the development and implementation of standards using patented technologies, the field of synthetic biology could benefit from the lessons learned by the information and communications technologies (ICT) industry, where a multitude of patented technologies have been incorporated into standards [51]. There, the creation of standards development organizations with formal policies requiring fair, reasonable and non-discriminatory (F/RAND) licensing terms have helped to alleviate some of the problems the ICT industry has faced in incorporating patented technologies into standards [52]. Because the creation of a standards development organization is not a trivial undertaking and could potentially raise antitrust concerns, it will be important to work with counsel and abide by the recommendations of governmental agencies [53,54].

Several limitations should be taken into account when interpreting the results from this survey. First, this survey sampled only a fraction of the global synthetic biology research community and included responses from 137 individuals, mostly from universities or research institutes within the United States. It is difficult to estimate the number of individuals conducting research in synthetic biology, though one study identified nearly 3,000 authors of scientific publications who were working on or writing about synthetic biology [55]. Given the relatively small sample size, the survey results may be subject to sampling bias (i.e., the demographics of the survey respondents may not accurately reflect the demographics of the synthetic biology research community) as well as potential reporting biases (i.e., the voluntary reporting of this survey necessarily excludes those who chose not to volunteer responses). A larger sampling of the synthetic biology research community, with greater representation of researchers outside of the United States as well as researchers working in industry, might provide more balanced insight into the technologies considered enabling for synthetic biology. Second, although in-person interviews were conducted to identify relevant technologies and create questions for the survey, the majority of responses were obtained through an online format. Without an interactive format, such as in-person or telephone interviews, to clarify any ambiguities in the wording of survey questions, respondents may have misunderstood some of the questions posed. Third, the survey questions focused on the technologies actually used by the synthetic biology research community and did not explore the potential reasons underlying why certain technologies were not used. Additional questions that directly query whether intellectual property rights covering certain technologies represented a selection barrier against the use of those technologies would also be informative. Finally, the results of this survey reflect the experiences of synthetic biology researchers at only one point in time. Re-administration of surveys such as the one developed here could provide a more complete view of the technologies that are considered enabling for synthetic biology as the field develops over time.

Beyond facilitating the systematic investigation of property rights, monitoring the enabling technologies of synthetic biology could also help inform governmental and non-governmental organizations in crafting policy frameworks to address the safety and security concerns raised by innovation in this field. Access to concrete data on the technologies used by those working in basic and applied synthetic biology research can be vital for making changes to existing policies as well as for creating new options for governance. For example, advances in DNA synthesis technology and the resulting commercial availability of larger synthetic DNA constructs [56] have led to a shift from research conducted with recombinant DNA to research conducted with synthetic nucleic acid molecules. This shift in the technologies used for research, in turn, has prompted the U.S. Department of Health and Human Services to issue amended guidelines for research involving recombinant or synthetic nucleic acid molecules [57] and to develop recommendations for a framework for synthetic nucleic acid screening [58]. Similarly, monitoring the enabling technologies of synthetic biology could help alert policy makers and stakeholders to advances in technology that may exert a comparable impact on innovation and research practices in this field.

## Conclusion

The survey results presented here provide insight into the enabling technologies of synthetic biology. As innovation in this field continues to advance we expect that the reagents, methods, and tools considered enabling for synthetic biology will change. The policies and practices of government, funding, and community organizations that impact the regulation, patenting, and licensing of these technologies are also subject to change. Because research in synthetic biology is conducted across multiple institutions in many countries, it will be important to adopt policies and practices that promote cross-institutional and transnational exchange of ideas, data, and technology. Moreover, because it is likely that useful applications of synthetic biology will embody multiple patented inventions, it will be important to create structures for managing intellectual property rights that will promote access to the technologies used to comprise and create commercially available products. By monitoring the enabling technologies of synthetic biology and advancing policies and best practices for the patenting, licensing and regulation of these technologies, our hope is that the field will be better able to reach its full potential to promote human health and preserve the environment.

## Methods

### Survey design, distribution and analysis

We designed a web-based survey soliciting responses on technologies that could be considered enabling by practitioners engaged in synthetic biology research. For purposes of the survey, enabling technologies were defined as tools, reagents, and methods that, alone or in combination with associated technologies, provide the means to generate any new research tool or application in synthetic biology. Technologies included in the survey were compiled through review of the scientific literature and personal interviews with synthetic biology researchers from both academia and industry. Researchers working in the field of synthetic biology were located by means of personal references, professional networking, and referrals from synthetic biology organizations. For this first survey, we focused on technologies used in research 1laboratories for the engineering of biological systems. Given its seminal importance in promoting a sense of community [59], we also examined the role of the iGEM competition in fostering the adoption of certain technologies by synthetic biology researchers. Technologies associated with safety and security were not within the scope of this survey, nor were other potentially enabling resources such as professional societies or technology roadmaps.

We first piloted the survey by sending the questionnaire to researchers working in the laboratories of Drs. Christina Smolke and Drew Endy in the Bioengineering Department at Stanford University, and made adjustments based on initial responses. We then sent the survey to members of the Synthetic Biology Engineering Resource Center (SynBERC) and made further adjustments based on responses received by Days 6 and 19. On Day 19 and at various times thereafter, a link to the survey was forwarded to additional researchers working in the field of synthetic biology by the BioBricks Foundation (BBF), a public benefit organization that represents the public interest in the field of synthetic biology (http://biobricks.org), the ERASynBio, a program for the development and coordination of synthetic biology in the European Research Area (http://www.erasynbio.eu), the iGEM Foundation, a public benefit organization that organizes the iGEM competition (http://igem.org), the organizers of SynBioBeta, an industry conference for synthetic biology startup companies (http://synbiobeta.com), and individuals working in community biolabs.

The survey was available via an interactive website (http://www.surveymonkey.com) or as a Word document directly from the authors, and the responses reported were collected from August 31, 2012 through January 30, 2013. Instructions provided at the beginning of the survey encouraged respondents to answer the questions based on their own experience and perspective. A PDF file of the survey questions is available as Additional file 1.

Questionnaire data were exported into Microsoft Excel for analysis and only valid responses were evaluated (i.e., only responses to the specific questions were included in each analysis). Analyses that involved comparison of researchers in academia and industry included only responses from survey participants that worked exclusively in a non-commercial or commercial setting. Survey participants were considered to be working in a non-commercial setting if they indicated that they worked in a college or university, research institute, government laboratory, or were independent (e.g., citizen scientist, amateur biologist, etc.). Survey participants were considered to be working in a commercial setting if they indicated that they worked in a for-profit company of any size. Statistical significance was evaluated using Fisher’s exact test over binary contingency tables [60].

### Registries of natural or engineered biological materials or information

Eleven publicly available registries were listed in Question 3 throughout the duration of the survey: the iGEM Registry (http://partsregistry.org), the JBEI-ICE Public (https://public-registry.jbei.org), the SynBERC Registry (https://registry.synberc.org), Addgene (http://www.addgene.org), the DNASU Plasmid Repository (http://dnasu.asu.edu/DNASU/Home.jsp), the DF/HCC PlasmID Repository (http://plasmid.med.harvard.edu/PLASMID), the ATCC (http://www.atcc.org), the CGSC (http://cgsc.biology.yale.edu), the EUROSCARF (http://web.uni-frankfurt.de/fb15/mikro/euroscarf/index.html), the Félix d’Hérelle Reference Center for Bacterial Viruses (http://www.phage.ulaval.ca/no_cache/en/accueil), and the ARS/NRRL culture collection (http://nrrl.ncaur.usda.gov). This group of publicly available registries includes both registries that distribute tangible materials (e.g., cell lines, plasmids, DNA binding proteins) and registries that serve solely as repositories of information (e.g., DNA sequences, plasmid construction, performance specifications). Two additional publicly available registries were added to the list for Question 3 after the collection of 85 responses, based on free text responses – the BCCM (http://bccm.belspo.be/index.php) and the DSMZ (http://www.dsmz.de).

### Physical assembly standards and methods

Sixteen physical assembly standards and methods were listed in Question 6 throughout the duration of the survey: the original BioBrick assembly standard (BBF RFC 10) [61], the BglBrick assembly standard (BBF RFC 21) [62], the BioFusion standard (BBF RFC 23) [63], Freiberg standard (BBF RFC 25) [64], the AarI cloning standard (BBF RFC 28) [65], the BioBytes assembly standard (BBF RFC 47) [17], Circular Polymerase Extension Cloning (CPEC) [66], DNA assembler [67], Gateway recombinatorial cloning [68], Gibson assembly [40], GoldenBraid assembly [69], GoldenGate shuffling [70], Modular Cloning (MoClo) [71], Seamless Ligation Cloning Extract (SLICE) [72], Sequence and Ligase Independent Cloning (SLIC) [73], and *de novo* DNA synthesis [74]. Two additional physical assembly methods were added to the list in Question 6 after the collection of 52 responses based on free text responses – Polymerase Incomplete Primer Extension (PIPE) [75] and Uracil Specific Excision Reagent (USER) [76].

### Tools for measurement, functional composition and data exchange

Tools supporting functional composition of genetic objects, measurement of intracellular molecular activities, and data exchange were listed in Survey Questions 7 and 8. Measurement tools included Polymerase Per Second (PoPS) [77], relative promoter unit (RPU) [47], relative mammalian promoter unit (RMPU) [78], functional composition tools included the expression operating unit (EOU) [45], and data exchange tools included Synthetic Biology Open Language (SBOL) [79], SBOL Visual (SBOLv) [80,81], and visual or electronic data sheets for biological parts and devices [82,83].

### Additional tools, methods and reagents

Additional tools, reagents, and methods that survey participants could consider enabling were listed in Survey Question 9. Genomic databases included the DNA Databank of Japan (http://www.ddbj.nig.ac.jp), European Nucleotide Archive (http://www.ebi.ac.uk/ena), GenBank (http://www.ncbi.nlm.nih.gov/genbank), e!EnsemblGenomes (http://www.ensemblgenomes.org), and MicrobesOnline (http://www.microbesonline.org). Tools for searching, alignment, and analysis of DNA sequences such as BLAST [84], ClustalW2 [85], and OligoCalc [86], respectively, as well as commercial and in-house methods for DNA synthesis [74] and DNA sequencing [87] were also included. Long-established cell culture technologies included antibiotic selection, temperature selection, lysogeny broth (a.k.a., Luria-Bertani medium or LB medium) [37,88], colorimetric media [89], and glycerol freezing of bacterial strains [38,39]. More recently established tools such as PCR [10,11], fluorescent reporter molecules [90], and directed evolution [91] were also included.

### Software tools

Thirty software tools, many of which have been recently reviewed [92], were listed in Question 10 throughout the duration of the survey: ApE (http://biologylabs.utah.edu/jorgensen/wayned/ape), BioJADE (http://web.mit.edu/jagoler/www/biojade), BioNetCAD (http://www.sysdiag.cnrs.fr/BioNetCAD), Cell Designer (http://celldesigner.org), ClothoCAD (http://www.clothocad.org), COPASI (http://www.copasi.org), DeviceEditor (http://replaced by AutoBioCAD; http://j5.jbei.org/index.php/Main_Page), Eugene (http://eugenecad.org), GEC (http://research.microsoft.com/en-us/projects/gec), Gene Designer (https://www.dna20.com/genedesigner2), GeneDesign (http://www.genedesign.org), GenoCAD (http://www.genocad.org), Genetdes (http://jaramillolab.issb.genopole.fr/display/sbsite/Download), GLAMM (http://glamm.lbl.gov), iBioSim (http://www.async.ece.utah.edu/iBioSim), j5 DNA Assembly Design Automation Software (http://j5.jbei.org/index.php/Main_Page), Mathematica (http://www.wolfram.com/mathematica), Mfold (http://mfold.rna.albany.edu/?q=mfold), OptCircuit (http://maranas.che.psu.edu/research_circuits.htm), Primer3 (http://simgene.com/Primer3), ProMoT (http://www.mpi-magdeburg.mpg.de/projects/promot), ProtoBiocompiler (http://proto.bbn.com/Proto/Proto.html), RBS Calculator (https://salis.psu.edu/software), Rosetta (http://www.rosettacommons.org), RoVerGeNe (http://iasi.bu.edu/~batt/rovergene/rovergene.htm), SimBiology - MATLAB (http://www.mathworks.com/products/simbiology), SynBioSS (http://www.synbioss.org), TinkerCell (http://www.tinkercell.com), VectorEditor (http://j5.jbei.org/index.php/Main_Page), and Vector NTI (http://www.invitrogen.com/site/us/en/home/Products-and-Services/Applications/Cloning/vector-nti-software.html). Three additional software tools were added to the list for Question 10 after the collection of 51 responses based on free text responses – GenomeCompiler (http://www.genomecompiler.com), GENtle (http://gentle.magnusmanske.de), and SnapGene (http://www.snapgene.com).

## Abbreviations

ARS/NRRL: Agricultural research service NRRL culture collection; ATCC: American type culture collection; BBF: BioBricks foundation; BCCM: Belgian coordinated collections of micro-organisms; BIOFAB: BIOFAB international open facility advancing biotechnology; CGSC: Coli genetic stock center; DF/HCC: Dana-farber/harvard cancer center; DSMZ: Leibniz-Institut DSMZ - German collection of microorganisms and cell cultures; EOU: Expression operating unit; EUROSCARF: European *Saccharomyces cerevisiae* archive for functional analysis; GFP: Green fluorescent protein; iGEM: International genetically engineered machines; JBEI-ICE Public: Joint BioEnergy institute public registry; LB medium: Luria-bertani medium; MoClo: Modular cloning; PIPE: Polymerase incomplete primer extension; PoPS: Polymerase per second; RBS: Ribosome binding site; RMPU: Relative mammalian promoter unit; RPU: Relative promoter unit; SLIC: Sequence and ligase independent cloning; SLICE: Seamless ligation cloning extract; SynBERC: Synthetic biology engineering resource center; SBOL: Synthetic biology open language; SBOLv: SBOL visual; SMBL: Systems biology markup language; USER: Uracil specific excision reagent.

## Competing interests

DE is a co-founder and director of a commercial DNA assembly company (Gen9, Inc.). No other competing interests have been declared by the authors.

## Authors’ contributions

LJK and DE conceived the study. LJK designed the survey, analyzed the responses, and wrote the manuscript. DE provided critical feedback and suggestions during the development and analysis of the survey. Both authors reviewed and approved the final manuscript.

## Supplementary Material

Additional file 1 PDF file of the survey questions. Click here for file
