# Supplementary material for: A survey of enabling technologies in synthetic biology
Source: J Biol Eng. 2013 May 10;7:13. doi: 10.1186/1754-1611-7-13 (PMC3684516; doi:10.1186/1754-1611-7-13)
Supplement: Additional file 1 — PDF file of the survey questions. [file 1754-1611-7-13-S1.pdf]

Enabling Technologies in Synthetic Biology

Dear Colleagues,

Please allow me to introduce myself. I am a research associate working with Drew Endy to investigate and develop options for how property rights can best be applied and adapted to support innovation in synthetic biology. As part of my work, I would like to assemble a baseline list of the "enabling technologies" of synthetic biology. This would be a helpful first step in evaluating the background property rights status for the technologies that are considered most useful/valuable for research and development of synthetic biology applications.

Please help us to define the enabling technologies of synthetic biology by completing this survey. The survey should take no more than 10 minutes to complete - just answer the questions based on your own experience and perspective. EVERYONE WHO SENDS IN A RESPONSE WITH THEIR EMAIL ADDRESS WILL RECEIVE A SUMMARY OF THE RESULTS.

Thank you in advance for your time and consideration. Please don't hesitate to call or email with any questions or even just to chat about enabling technologies in synthetic biology.

With much appreciation!
Linda Kahl, PhD, JD
lkahl@stanford.edu

P.S. Please forward this link to other synthetic biology researchers in academics, research institutions, community groups and industry!

1. Please indicate the sector(s) in which you currently work:

College or University

Research Institution

Public Benefit Organization

Independent (citizen scientist, amateur biologist, etc.)

For-profit company (<50 employees)

For-profit company (<250 employees)

For-profit company (<1000 employees)

For-profit company (>1000 employees)

Other (please specify)

2. Please indicate your experience with the International Genetically Engineered Machines (iGEM) competition:

Please indicate any other experience you may have with iGEM (for example, as a team leader):

3. Please indicate whether you use biological parts from, or contribute parts to, the following registries:

	Yes, I've used parts from this registry	Yes, I've contributed parts to this registry
Registry of Standard Biological Parts (http://partsregistry.org)		
JBEI-ICE Public (https://public-registry.jbei.org)		
SynBERC Registry (https://registry.synberc.org)		
Addgene (http://www.addgene.org)		
BIOFAB (http://io.biofab.org/services/studio/dac)		
DNASU Plasmid Repository (http://dnasu.edu)		
DF/HCC PlasmID Repository (http://plasmid.med.harvard.edu/PLASMID)		
ATCC (http://www.atcc.org)		
CGSC (http://cgsc.biology.yale.edu)		
EUROSCARF (http://web.uni-frankfurt.de/fb15/mikro/euroscarf/index.html)		
Félix d'Hérelle Reference Center for Bacterial Viruses		

(http://www.phage.ulaval.ca/no_cache/en/accueil)

NRRL Collection (aka ARS Culture Collection) (http://nrrl.ncaur.usda.gov)

Belgian Co-ordinated Collections of Micro-organisms (http://bccm.belspo.be/index.php)

Leibniz-Institut DSMZ - German Collection of Microorganisms and Cell Cultures (http://www.dsmz.de)

Please list the name and URL (if available) of any other registry of which you are aware:

4. Please indicate whether you use a private registry of biological parts.

	Yes	No
Does the lab in which you work maintain its own registry of biological parts?		
Are some or all of these parts made available to others outside the lab (upon publication, for example)?		
Does the lab send parts to others directly?		
Does the lab distribute parts through a public registry (such as those listed in question 3 above)?		

5. Please list your favorite/most useful biological parts (for example, promoter BBa_J23101 from the Registry of Standard Biological Parts)

6. Please indicate whether you use any of the following physical assembly standards or methods:

	I currently use this.	I've used this is the past.	I don't use this.
BioBrick assembly standard (BBF RFC 10)			
BglBrick assembly standard (BBF RFC 21)			

BioFusion standard (BBF RFC 23)

Freiberg standard (BBF RFC 25)

AarI cloning standard (BBF RFC 28)

BioBytes assembly standard (BBF RFC 47)

Circular Polymerase Extension Cloning - CPEC (Quan & Tian 2009)

Conventional restriction site-based cloning (e.g. home-brew PCR)

DNA Assembler (Shao et al. 2009)

Gateway recombinatorial cloning (Hartley et al. 2000)

Gibson Assembly (Gibson et al. 2009)

GoldenBraid (Sarrion-Perdigones et al. 2011)

GoldenGate Shuffling (Engler et al. 2008)

MoClo (Weber et al. 2011)

Polymerase Incomplete Primer Extension - PIPE (Klock & Lesley, 2009)

Restriction-site Associated DNA (RAD) assembly (Etter et al. 2011)

Seamless Ligation Cloning Extract - SLICE (Zhang et al. 2012)

Sequence and ligase independent cloning - SLIC (Li & Elledge 2007)

Uracil Specific Excision Reagent - USER (Bitinaite et al. 2007)

Yeast in vivo recombinational cloning (Oldenburg, 2007)

de novo DNA synthesis (reviewed in Carr & Church 2009)

Please list any other assembly method (commercial or home-brew) that you use:

7. Please indicate whether you use any of the following measurement tools or methods:

	I currently use this.	I've used this in the past.	I don't use this.
Polymerase Per Second (PoPS) (Endy 2005)			
Relative Promoter Unit (BBF RFC 19) (Kelley et al. 2009)			
Relative Mammalian Promoter Unit (BBF RFC 41)			
Expression Operating Unit (Mutalik et al. 2012)			

Please list any other measurement tools or methods that you use:

8. Please indicate whether you use any of the following data exchange tools:

	I currently use this.	I've used this in the past.	I don't use this.
Synthetic Biology Open Language (SBOL) (BBF RFC 87)			
SBOL visual (SBOLv) (BBF RFC 68)			
JBEI-ICE Repository Platform (Ham et al. 2012)			
Visual Datasheets (for examples see Canton et al. 2008, Lee et al. 2011)			
Electronic Datasheets (for example http://biofab.org/data)			

Please list any other data exchange tool you use:

9. Please indicate whether you use any of the following tools, reagents or methods:

	I currently use this	I've used this in the past	I don't use this
DNA Databank of Japan (http://www.ddbj.nig.ac.jp/)			
European Nucleotide Archive			

(http://www.ebi.ac.uk/ena/)

GenBank
(http://www.ncbi.nlm.nih.gov/genbank/)

e!EnsemblGenomes
(http://www.ensemblgenomes.org/)

MicrobesOnline
(http://www.microbesonline.org/)

Sequence search tools (e.g., BLAST)

Sequence alignment tools (e.g. ClustalW2)

Sequence analysis tools (e.g., OligoCalc)

Directed evolution technologies (e.g., MAGE)

in-house DNA synthesis - short oligonucleotides

commercial DNA synthesis - short oligonucleotides

in-house DNA synthesis - gene size pieces (>500 bp)

commercial DNA synthesis - gene size pieces (>500 bp)

in-house DNA sequencing

commercial DNA sequencing

Polymerase chain reaction

LB broth or agar

Colorimetric, solid culture medium (e.g., TK medium)

Glycerol freezing of bacteria

Antibiotic selection

30*C incubator

37*C incubator

Green Fluorescent Protein

non-GFP reporter molecules

Please list any reagents or methods that you consider to be an "enabling technology" in synthetic biology. Enabling technology = reagent or method that, alone or in combination with

associated technologies, provides the means to generate any new research tool or application in synthetic biology.

10. Please indicate whether you use any of the following software tools:

	I currently use this.	I've used this in the past.	I don't use this.
APE, A Plasmid Editor (http://biologylabs.utah.edu/jorgensen/wayned/ape/)			
BioJADE (http://web.mit.edu/jagoler/www/biojade/)			
BioNetCAD (http://www.sysdiag.cnrs.fr/BioNetCAD)			
BLAST (http://blast.ncbi.nlm.nih.gov)			
Cell Designer (http://celldesigner.org)			
CLC Genomics Workbench (http://www.clcbio.com/products/clc-genomics-workbench)			
ClothoCAD (http://www.clothocad.org)			
COPASI (http://www.copasi.org)			
DeviceEditor (http://j5.jbei.org/index.php/Main_Page)			
Eugene (http://eugenecad.org)			
GEC (http://research.microsoft.com/en-us/projects/gec/)			
Gene Designer (https://www.dna20.com/genedesigner2)			
GeneDesign (http://www.genedesign.org)			
GenoCAD (http://www.genocad.org)			
Geneious (http://www.geneious.com)			
Genetdes (http://synth-bio.yi.org/genetdes.html)			
GenomeCompiler (http://www.genomecompiler.com/)			
GENtle (http://gentle.magnusmanske.de/)			
GLAMM (http://glamm.lbl.gov/)			

iBioSim (http://www.async.ece.utah.edu/iBioSim)

j5 DNA Assembly Design Automation Software (http://j5.jbei.org/index.php/Main_Page)

Lasergene-DNAStar (http://www.dnastar.com)

Mathematica (http://www.wolfram.com/mathematica)

Mfold (http://mfold.rna.albany.edu/?q=mfold)

OptCircuit (Dasika & Maranas, 2008)

Primer3 (http://simgene.com/Primer3)

ProMoT (http://www.mpi-magdeburg.mpg.de/projects/promot)

ProtoBiocompiler (http://proto.bbn.com/Proto/Proto.html)

RBS Calculator (https://salis.psu.edu/software)

Rosetta (http://www.rosettacommons.org)

RoVerGeNe (http://iasi.bu.edu/~batt/rovergene/rovergene.htm)

SimBiology - MATLAB (http://www.mathworks.com/products/simbiology)

SnapGene (http://www.snapgene.com/)

SynBioSS (http://www.synbioss.org)

TinkerCell (http://www.tinkercell.com)

VectorEditor (http://j5.jbei.org/index.php/Main_Page)

Vector NTI (http://www.invitrogen.com/site/us/en/home/Products-and-Services/Applications/Cloning/vector-nti-software.html)

Please list the name and URL (if available) of any other software tool you find useful:

11. Do you consider yourself to be a synthetic biologist, or to be engaged in basic or applied synthetic biology research or development?

Yes

No

Please comment on your background in synthetic biology

12. What is your "wish list" for technologies that you would like to see developed (or developed further) in the future?

13. Please specify the country in which you conduct the majority of your work in synthetic biology.

Country:

14. Please specify your current college/university or place of employment:

15. Please indicate your current position:

Student, undergraduate

Student, graduate

Postdoctoral fellow, academics

Postdoctoral fellow, industry

University Faculty

University Staff

Research & Development Scientist/Engineer

Process Scientist/Engineer

Computational Scientist/Engineer

Other (please specify)

16. Please use the space below to offer any comments, suggestions, etc.

17. (OPTIONAL) Please list your name and email address

EVERYONE WHO SENDS IN A RESPONSE WITH THEIR EMAIL ADDRESS WILL RECEIVE A SUMMARY OF THE RESULTS!

(names and email addresses will be kept confidential)

Done

Powered by **SurveyMonkey**
Check out our sample surveys and create your own now!
